# Electrocatalytic Hollow AgAu/SiO_2_ Sensor for Multi-Matrix Isoproturon Monitoring

**DOI:** 10.1021/acsomega.5c02480

**Published:** 2025-08-12

**Authors:** Jose Antonio de Oliveira Junior, Antonio Gomes dos Santos Neto, Camila Silva de Sousa, Rebeca Yatsuzuka, Felipe Anchieta e Silva, Thenner Silva Rodrigues, Marco Aurélio Suller Garcia, Cristiane Luisa Jost, Sergio Yesid Gomez Gonzalez

**Affiliations:** † Departamento de Engenharia Química e Engenharia de Alimentos, Universidade Federal de Santa Catarina, Campus Universitário Trindade, 88040-900 Florianópolis, SC, Brazil; ‡ Ampere-Laboratório de Plataformas Eletroquímicas, Departamento de Química, 28117 Universidade Federal de Santa Catarina, Campus Universitário Trindade, 88040-900 Florianópolis, SC, Brazil; § Central Analítica, Instituto de Química, Universidade de São Paulo, Av. Prof. Lineu Prestes, 748, 05508-000 São Paulo, SP, Brazil; ∥ Programa de Engenharia de Nanotecnologia, Instituto Alberto Luiz Coimbra de Pós-Graduação e Pesquisa em Engenharia, COPPE, Universidade Federal do Rio de Janeiro, Av. Horácio Macedo, 2030, 21941-972 Rio de Janeiro, RJ, Brazil; ⊥ Departamento de Química, Universidade Federal do Maranhão, Av. dos Portugueses,1966, 65080-805 São Luis, MA, Brazil

## Abstract

This study reports the development and application of an electrochemical sensor based on a glassy carbon electrode (GCE) modified with hollow AgAu nanoshells supported on SiO_2_ (AgAu NSs/SiO_2_) and Nafion as a binder. The proposed AgAu NSs/SiO_2_/NF/GCE sensor demonstrated excellent analytical performance for detecting isoproturon (ISO), a persistent environmental contaminant of significant concern. The sensor exhibited a wide linear detection range from 0.75 to 20 μg L^–1^, a low detection limit of 0.011 μg L^–1^, and a quantification limit of 0.037 μg L^–1^, meeting and exceeding regulatory requirements. Differential pulse voltammetry revealed a remarkable 220% increase in peak current response compared to the unmodified electrode, indicating superior electrocatalytic activity. The sensor also demonstrated high repeatability (RSD = 3.28%), reproducibility (RSD = 1.10%), and excellent selectivity with minimal interference from complex matrices. Its applicability was successfully validated in aquaculture water, tomato extract, and human plasma samples, achieving recovery rates ranging from 83.0 to 100.4%. The key contribution of this work is the synthesis of AgAu nanoshells via a galvanic replacement reaction and their integration into an electrochemical sensor, which exhibits enhanced sensitivity, selectivity, and environmental applicability. This novel sensing platform offers a practical, efficient, and versatile tool for ISO monitoring across diverse real-world samples.

## Graphical abstract

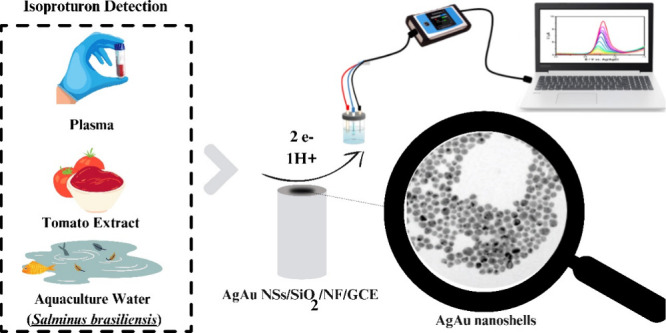

## Introduction

Phenylurea pesticides have garnered substantial attention due to their persistence and widespread distribution in environmental matrices. Among these compounds, ISO stands out as a critical concern, frequently detected in soil,^2^ aquatic ecosystems,^3^ food products such as tomato extracts,^1,2^ and even human biological samples, including blood plasma;^3^ The accumulation of ISO not only exacerbates ecological risks—such as the decline of biodiversity and the emergence of herbicide-resistant weed populations—but also underscores the urgent need for advanced monitoring tools capable of addressing its pervasive presence and potential health impacts.^4^

Such behavior is linked to its production, transportation, storage, utilization, accidental spills, and the washing of spraying equipment. These activities result in ISO concentrations in surface and groundwater that, in some cases, exceed the maximum allowable limit of 0.10 μg L^–1^ set by the European Commission’s Water Framework Directive and the default lowest limit of analytical determination value of 0.01 mg kg^–1^ when a pesticide is not explicitly mentioned (6,7). Excessive exposure can affect the bloodstream and is acutely toxic to algae and oysters at concentrations exceeding 13 and 370 μg L^–1^, respectively.^5^ Additionally, fish growth is inhibited at concentrations greater than 1.0 mg L^–1^.^5^ Therefore, their prolonged environmental presence raises sustainability challenges, necessitating effective monitoring and management strategies. Thus, assessing the presence and quantity of ISO is crucial, and sensitive and selective detection methods are indispensable.

The critical importance of early phenylurea detection cannot be overstated, as timely identification enables effective mitigation of both environmental contamination and public health risks. Current regulatory frameworks (including EPA Method 535.1 and EU Directive 2008/105/EC) designate liquid chromatography–tandem mass spectrometry (LC–MS/MS) as the reference technique. For phenylureas, high-performance liquid chromatography (HPLC)^6–9^ and gas chromatography–mass spectrometry (GC–MS)^10–12^ are often used approaches. While these methods offer exceptional sensitivity and reliability for laboratory analysis, their field deployment faces fundamental constraints such as substantial capital costs and demanding sample preparation protocols. These limitations have driven the development of innovative field-deployable alternatives, particularly electrochemical sensors incorporating nanomaterials, which provide distinct advantages for environmental monitoring.

Electrochemical sensors have then become recognized as an up-and-coming technology in analytical instrumentation.^13–21^ Their rapid response times enable quick measurements, which is crucial for time-sensitive applications.^22,23^ Additionally, these sensors are cost-effective, making them accessible for widespread use across various activities, including environmental monitoring, healthcare, and food safety.^24,25^ Moreover, the versatility of electrochemical sensors, which can be adapted and tailored for different targets, further highlights their potential as an essential tool for a broad range of analytical applications.^26^ In this scenario, the use of noble metals in electrochemical sensing is generally beneficial due to their high catalytic activity, stability, conductivity, and biocompatibility.^27^ These properties make them ideal for sensitive and reliable measurements, particularly in environments that require durability and precise detection.^28^

Gold (Au) is particularly valuable in electrochemical sensing due to its excellent conductivity and exceptional oxidation and corrosion resistance, making it highly stable under various testing conditions.^29^ This stability is crucial for applications where sensors are exposed to harsh environments.^30^ However, its high cost and limited availability are significant drawbacks. In this sense, nanotechnology significantly enhances the utilization of expensive noble metals by increasing their efficiency and reducing the quantities required.^31,32^ Thus, by manipulating materials at the nanoscale, it is possible to tailor nanostructures by controlling their size, shape, and composition.^33,34^

Interestingly, galvanic replacement stands out among several nanomaterials’ syntheses due to its low utilization of noble metals, particularly when a sacrificial template is used, such as silver (Ag).^35^ When the synthesis method is well-performed, it generates hollow structures. In electrochemical processes, these materials offer a higher surface area-to-volume ratio than their solid counterparts, thereby enhancing sensitivity by allowing for more extensive interaction with target molecules. The hollow interiors improve catalytic activity and facilitate the diffusion of analytes and electrolytes, which is beneficial for rapid detection applications.^36–38^ Furthermore, electrocatalytic applications have demonstrated that the randomness of metal disposition induced by the synthesis process can be advantageous.^39,40^ Such a phenomenon merits further exploration in the field of sensing.

Gold–platinum bimetallic nanoparticles (AuPtNPs) have shown considerable promise as electrode modifiers due to their synergistic catalytic and conductive properties.^41^ Previous studies have demonstrated their effectiveness in high peroxidase-like activity and H_2_O_2_ detection, for instance, by leveraging platinum’s catalytic activity and gold’s stability in electrochemical environments. SiO_2_ nanoparticles have also been strategically incorporated as a support material to optimize the sensor’s performance.^42^ The mesoporous structure of SiO_2_ (22 Å pore size, 800 m^2^/g surface area) not only prevented aggregation of AgPt nanotubes but also facilitated their uniform distribution, thereby maximizing active sites for ISO oxidation. Additionally, previous studies by some of us have proposed that the incorporation of Ag atoms in combination with noble-metal atoms through a galvanic replacement process promotes the formation of oxygen vacancies, thereby enhancing the material’s electrical conductivity.^43,44^

In this context, developing an electrochemical sensor based on AgAu nanoshells (NSs) supported on a glassy carbon electrode (GCE) using Nafion (NF) as binder – AgAu NSs/SiO_2_/NF/GCE – presents a novel and promising solution for ISO detection. Therefore, this paper presents the comprehensive development and characterization of the AgAu NSs/SiO_2_/NF/GCE sensor for detecting ISO in aquaculture water, tomato extract, and human plasma samples. This sensor offers a practical and effective solution for sensitive and selective ISO detection through rigorous electrochemical analyses and optimization, thereby contributing to enhanced environmental, food, and health monitoring.

## Materials and Methods

### Chemicals and Solutions

In this research, all the reagents used were of exceptional analytical quality. The chemicals included Isoproturon in PESTANAL grade (>99%), Nafion from Sigma-Aldrich, formic acid (Neon, 85%), ethanol (Merck, ≥99%), methanol (Merck, ≥99%), hydrochloric acid (HCl, 37%), boric acid (Vetec, 95%), acetic acid (Vetec, 95%), phosphoric acid (Vetec, 95%), citric acid (Vetec, 95%), and sodium hydroxide (Merck, ≥99%). For synthesizing the required materials, we utilized chloroauric acid trihydrate (HAuCl_4_·3H_2_O, 99.9%), silver nitrate (AgNO_3_, 99%), ethylene glycol (EG, 99.8%), polyvinylpyrrolidone (PVP) with molecular weights of 10,000 and 55,000 g mol^–1^, hydrochloric acid (HCl, 37%), and silica with a pore size of 22 Å and a surface area of 800 m^2^ g^–1^, all acquired from Sigma-Aldrich. Ultrapure water, obtained from a Milli-Q system (Millipore, Bedford, USA), with a resistivity of approximately 18.2 MΩ cm, was used to prepare all aqueous solutions.

To prepare ISO stock solutions with a concentration of 0.01 mg L^–1^, a solvent mixture of ethanol and deionized water in equal parts was used. To achieve lower concentrations, dilutions were made as necessary. The Britton-Robinson (BR) supporting electrolyte was prepared using a combination of acetic, boric, and phosphoric acids, each at a concentration of 0.1 mol L^–1^. The pH of the BR buffer was adjusted to a range of 2.0–8.0 by adding 0.25 mol L^–1^ NaOH. The McIlvaine supporting electrolyte was prepared by mixing monobasic sodium phosphate and citric acid to achieve a final concentration of 0.1 mol L^–1^. The phosphate buffer solution was prepared using 0.1 mol L^–1^ dibasic sodium phosphate, with its pH adjusted by adding saturated NaOH. The citrate buffer solution was made by adjusting a 0.1 mol L^–1^ citric acid solution with 0.25 mol L^–1^ NaOH to the desired pH. All solutions remained stable for 90 days when refrigerated. Standard solutions containing 1.0 g L^–1^ of lead (Pb^2+^), cadmium (Cd^2+^), zinc (Zn^2+^), and copper (Cu^2+^) ions were obtained from nitrate salts provided by SpecSol, São Paulo, Brazil. Various other potential interferents were included, such as diuron, pirimicarb, bisphenol, roxarsone, glyphosate, and carbofuran. Moreover, compounds such as ascorbic acid, uric acid, caffeic acid, and citric acid were also used in the experiment.

### Synthesis of AgAu NSs/SiO_2_

In a standard synthesis process, 5.0 g of PVP (10 000 g/mol) was dissolved in 37.5 mL of ethylene glycol (EG). Then, 200 mg of AgNO_3_ was added to this solution. Once completely dissolved, the solution was heated to 125 °C and maintained at this temperature for 2.5 h. The solution turned a greenish-yellow color and was allowed to cool to room temperature before being diluted to 125 mL with water. To prepare AgAu NSs, we mixed 5 mL of a 0.1 wt % aqueous PVP solution (55,000 g/mol) with 1 mL of the previously synthesized Ag nanospheres. This mixture was stirred for 10 min at 100 °C in a round-bottom flask. Then, dropwise, 2 mL of a 5 mM aqueous AuCl_4_^–^ solution was added. The reaction was then continued under stirring at 100 °C for 1 h. The immobilization of the AgAu NSs onto silica support was carried out using a wet impregnation method. Typically, a suspension of AgAu NSs was introduced into a beaker containing commercial silica and stirred continuously for 24 h at room temperature. Subsequently, the resultant solid was washed twice with water and twice with ethanol. After washing, the material was dried at 120 °C for 2 h in the air, producing the AgAu NSs/SiO_2_ material.^39^

### Physical Characterizations

Field-emission scanning electron microscopy (FEG-SEM) images were obtained using a JEOL field-emission gun electron microscope, JSM6330F (JEOL, Tokyo, Japan), operating at 5 kV. For imaging, samples were prepared by dissolving the material in an aqueous solution, which was then drop-cast onto a silicon wafer and allowed to dry under ambient conditions. Transmission electron microscopy (TEM) images were taken with an FEI TECNAI G2 F20 microscope (JEOL, Tokyo, Japan) operating at 200 kV. The sample was prepared by drop-casting an isopropanol solution containing the material onto a copper grid and dried under ambient conditions. The size distribution of the prepared nanoshells was determined by measuring 250 individual particles.

X-ray photoelectron spectroscopy (XPS) spectra were obtained using a Scienta Omicron ESCA+ spectrometer system, which features an EA 125 hemispherical analyzer and an XM 1000 monochromated X-ray source (Scientia Omicron, Uppsala, Sweden), utilizing Al Kα radiation (1486.7 eV). CasaXPS software version 2.3.15 (Casa Software Ltd., Teignmouth, United Kingdom) was employed for the spectral data analysis. The metal uptake onto the silica was quantified using inductively coupled plasma optical emission spectrometry (ICP-OES) conducted on Arcos equipment (SPECTRO Analytical Instruments, Kleve, Germany).

The ultraviolet–visible (UV–vis) spectrum was acquired using a Shimadzu UV-2600i spectrophotometer. For the sample preparation, 20 mg of the AgAu/SiO_2_ was dispersed in 100 mL of Milli-Q water to obtain a homogeneous suspension with a concentration of 0.2 g L^–1^. The suspension was sonicated in a sonication bath for 20 min to ensure complete dispersion. Subsequently, 1 mL of the prepared AgAu/SiO_2_ suspension was transferred to a quartz for spectral measurement across the wavelength range of 250–1000 nm.

The Fourier-transform infrared (FTIR) spectrum was recorded using an Agilent Cary 630 FTIR spectrometer. For the sample preparation, 20 mg of the AgAu/SiO_2_ was mixed with potassium bromide (KBr) and pressed into a transparent pellet. The measurement was performed in transmission mode, scanning the wavenumber region between 4000 and 500 cm^–1^.

### Electrochemical Analysis

A potentiostat/galvanostat system from PalmSens (Palm Instruments BV, Netherlands) was used to develop the voltammetric methodology. This system was connected to a computer running PSTrace software (version 5.5) for collecting and processing data. Several voltammetric techniques were applied, including cyclic voltammetry (CV), linear sweep voltammetry (LSV), differential pulse voltammetry (DPV), and square wave voltammetry (SWV).

The experimental procedure used a traditional three-electrode setup, including a 2.0 mm diameter glassy carbon working electrode (GCE), a platinum counter electrode, and an Ag/AgCl reference electrode saturated with KCl. Before each experiment, the GCE was meticulously polished with alumina powder (particle sizes: 1.00, 0.50, 0.30, and 0.05 μm from Buehler Ltd.), followed by thorough rinsing with deionized water and ethanol, and a 3 min sonication in distilled water to ensure the surface was clean. Voltammetric current data were derived from peak height measurements. The background currents were subtracted using PSTrace software version 5.8 in fixed baseline mode. All experiments were conducted at a stable room temperature of approximately 25 °C. Additionally, pH levels were determined using a pH meter (Ohaus ST3100-F), and reagents were dissolved with an UltraCleaner 800 ultrasonic cleaner.

Furthermore, solutions were stirred in the electrochemical cell with an IKA lab disc magnetic stirrer, and the reagents were precisely weighed on a Shimadzu Unibloc analytical balance (model AUX320) to prepare the solutions. For the pH analysis, the DPV technique was employed using a BR buffer over a pH range of 2.0–10.0, and the scan rate was investigated via CV at rates ranging from 10 to 100 mVs^–1^. Monolayer homogeneity and the number of electrons involved in the reaction were determined using the Laviron equation.^45^

### Preparation of Modified Electrodes

Before the tests, a suspension was prepared by mixing 2.5 mg of AgAu NSs/SiO_2_ with 0.5 mL of methanol, 0.02 mL of 5.00% Nafion, and 0.73 mL of deionized water. This mixture was then sonicated for 20 min. For the modification step, a predetermined volume of the AgAu NSs/SiO_2_/NF suspension, optimized via CV, was applied to the GCE. A 5 μL drop of the suspension was placed on the GCE, and the solvent was evaporated by heating at 50 °C for 15 min in an oven.

### Analytical Figures of Merit

A range of ISO standard solutions with different concentrations was prepared to establish the calibration curve. Aliquots from the stock solution were diluted to achieve the desired concentrations. Each diluted standard solution was measured three times using differential pulse voltammetry (DPV). The limits of quantification (LOQ) and detection (LOD) were calculated according to the IUPAC guidelines.^46^

To assess repeatability, five consecutive measurements were conducted on the same supporting electrolyte with ISO at a specific concentration within the linear range of the calibration curve.^46^ The mean current value was computed, and the relative standard deviation (RSD) of these measurements was analyzed. For reproducibility evaluation, the anodic peak current values of the ISO oxidation process were measured using five modified electrodes, all in the same supporting electrolyte with the analyte present. The RSD of these tests was then calculated according to eq 1:^46^

$$\%\mathrm{RSD}=S\times \frac{100}{X}$$ 1

where *S* refers to the standard deviation of measures and *X* is the mean average of those measurements.

### Real Sample Analysis

The developed method was applied to analyze real-world samples, including aquaculture water, tomato extract, and simulated plasma, showcasing its broad applicability across various matrices. To maintain sample integrity, all containers were autoclaved and prewashed before use. The aquaculture water sample was taken from Fazenda Experimental da Ressacada on February 19, 2024. This site is home to the endangered *Salminus brasiliensis* conservation stock of the Federal University of Santa Catarina, also known as the golden dorado, a species listed as endangered in the upper Uruguay River basins. During collection, critical parameters, including temperature (28.8 °C), pH (6.68), dissolved oxygen (73.8%), conductivity (71.4 μS/cm), and salinity (0.03 ppt), were precisely measured using the YSI Pro Plus water quality instrument. Human plasma and tomato extract samples were prepared following established procedures^47^

As utilized in previous works, the simulated human plasma sample was obtained from Sigma-Aldrich.^48^ In brief, plasma samples were diluted at a 1:10 ratio in a 0.1 mol L^–1^ BR supporting electrolyte with a pH of 3.0, and the same procedure was used for the tomato extract samples. Both types of samples were then sonicated for 5 min to ensure complete mixing. The DPV technique was used to determine the ISO concentration using the standard addition method under optimal conditions. Specifically, 1.00 mL of the enriched sample was introduced into the electrochemical cell, resulting in a final volume of 5.00 mL with the supporting electrolyte solution. Recovery rates were calculated according to the literature.^46^

## Results and Discussion

### Materials Physical Characterizations

A galvanic replacement process was performed to synthesize the material used in the sensing experiments. Initially, sacrificial-template Ag nanospheres were prepared using the polyol method with polyvinylpyrrolidone (PVP) as the stabilizer. According to the TEM image in Figure 1A, the nanospheres presented a diameter of 34 ± 2 nm. Then, [AuCl_4_]^−^(aq) ions were added to the solution, partially oxidizing the Ag nanospheres while reducing the Au species. The reaction that represents the process is

$${\mathrm{Ag}}_{\left(s\right)}+{{\left[{\mathrm{AuCl}}_{4}\right]}^{-}}_{\left(\mathrm{aq}\right)}\to {\mathrm{Au}}_{\left(s\right)}+3{{\mathrm{Ag}}^{+}}_{\left(\mathrm{aq}\right)}+4{{\mathrm{Cl}}^{-}}_{\left(\mathrm{aq}\right)}$$

**Figure 1:**
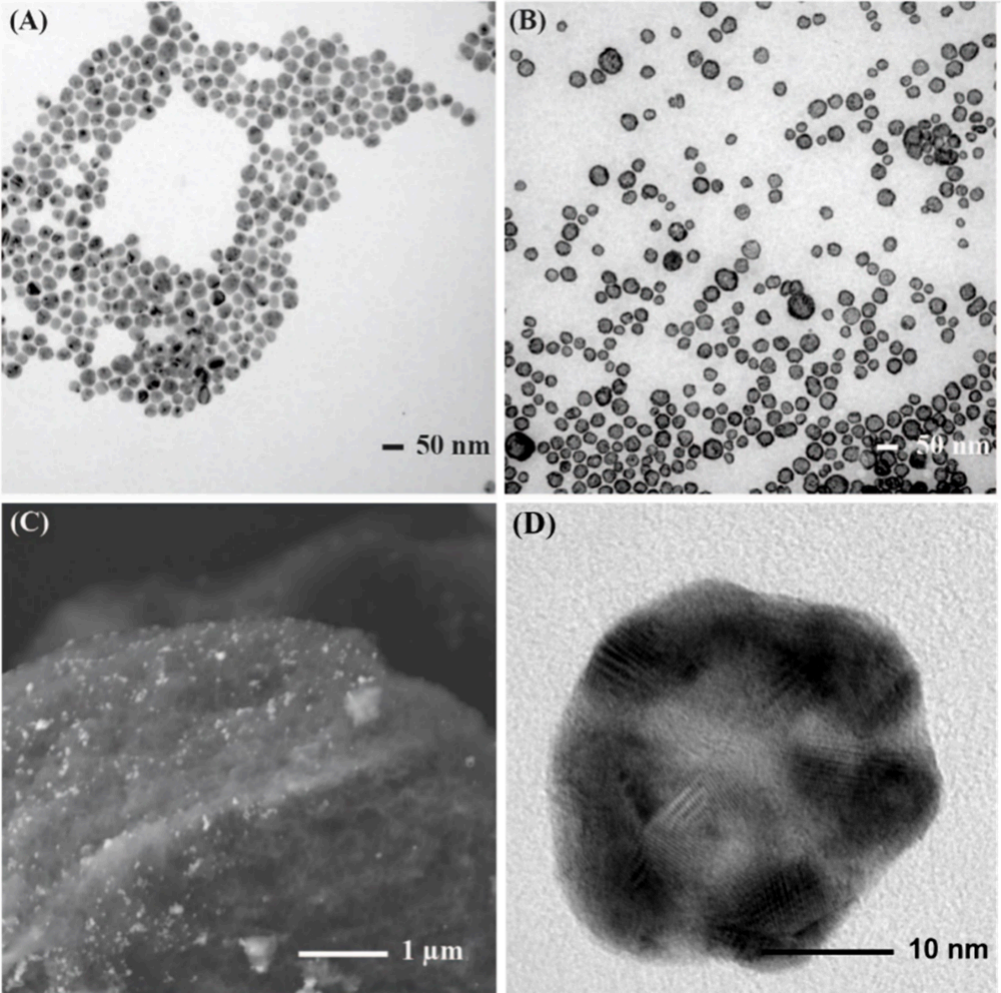
TEM images of (A) Ag nanosphere and (B) AgAu NSs. (C) SEM image of the AgAu NSs supported on commercial SiO_2_ (AgAu NSs/SiO_2_). (D) HRTEM of one AgAu nanoshell.

Such a procedure led to the formation of NSs (Figure 1B), which had a diameter of 37 ± 2 nm. This effect confirms the formation of NSs with a wall thickness of approximately 5 nm. SEM (Figure 1C) images revealed that these AgAu NSs, after immobilization, are uniformly distributed over the commercial SiO_2_; the as-prepared material is designated as AgAu NSs/SiO_2_. Additionally, ICP-OES analysis performed on the material used to obtain Figure 1 revealed a metal loading of 1.0 wt %, with approximately the same metal ratio for both metals. For further observations, it can be seen that Figure 1D reveals less dense interiors in the nanostructures due to the difference in mass–thickness contrast associated with the *Z* dependence of the image contrast, confirming the formation of hollow structures.

Figure 2A presents the Fourier-transform infrared (FTIR) spectrum of the AgAu/SiO_2_. FTIR spectroscopy enabled the investigation of surface functional groups and local structural characteristics across the spectral range of 4000–500 cm^–1^. The figure displays three principal vibrational features: (i) a broad absorption band centered at approximately 3400 cm^–1^, assigned to surface hydroxyl (−OH) groups and physisorbed water molecules; (ii) a distinct peak at 1620 cm^–1^, assigned to surface hydroxyl (−OH) groups and physisorbed water molecules; (ii) a distinct peak at 1620 cm^–1^, arising from the bending vibration of Si–OH groups (δ­(OH)) with potential contribution from H–O–H bending modes of adsorbed water; and (iii) a series of intense absorptions below 1400 cm^–1^ dominated by characteristic Si–O–Si vibrational modes.

**Figure 2:**
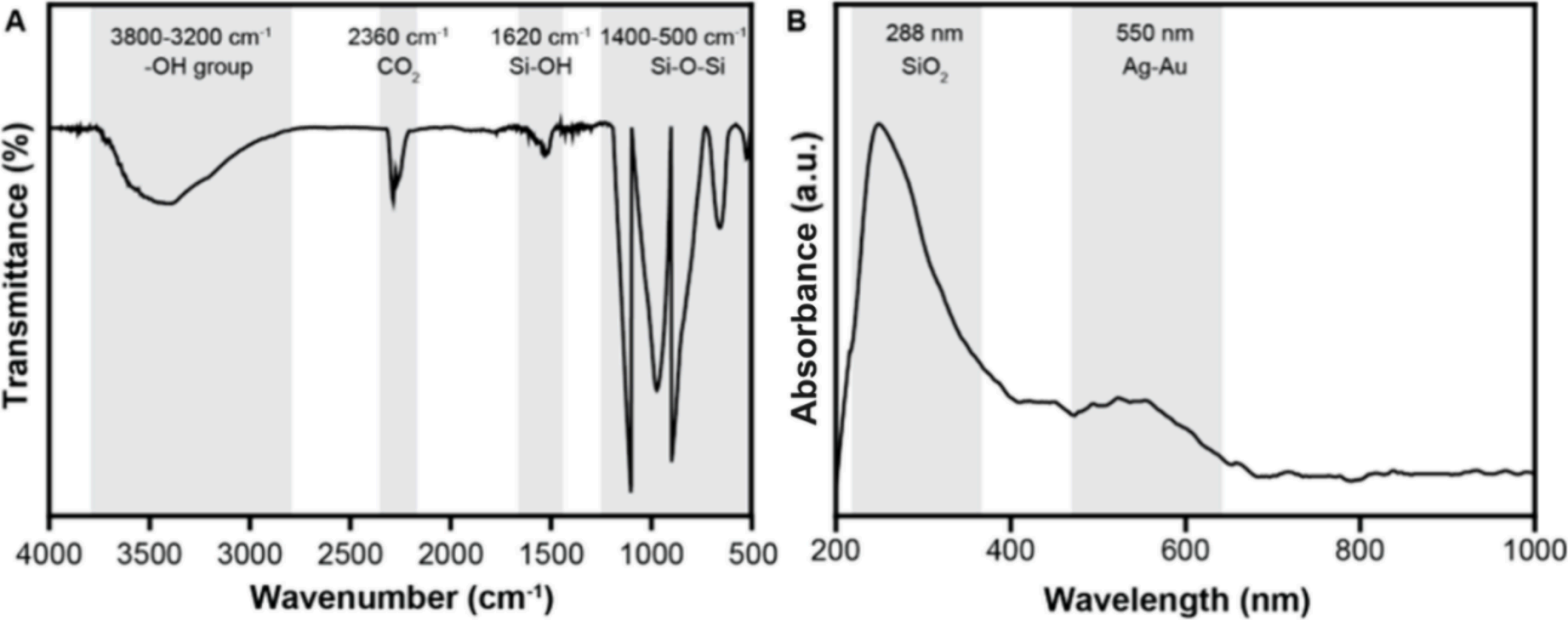
(A) FTIR and (B) UV–vis spectra of AgAu/SiO_2_.

The lower frequency region reveals four well-defined peaks between 1400–500 cm^–1^: the absorption at 1240 cm^–1^ corresponds to asymmetric stretching vibrations, while the strong doublet at 1110 and 1044 cm^–1^ represents network vibrations of the Si–O–Si framework. The features at 790 and 680 cm^–1^ are attributed to symmetric bending and rocking modes of Si–O–Si linkages, respectively. These spectral signatures are diagnostic of amorphous SiO_2_, with the particularly intense peak at 1110 cm^–1^ specifically indicating the asymmetric stretching vibration of bridging oxygen atoms in the cross-linked silica matrix. The spectrum additionally exhibits a doublet at 2360 and 2340 cm^–1^, characteristic of asymmetric stretching vibrations of atmospheric CO_2_ adsorbed during sample preparation. The absence of absorption bands corresponding to organic functional groups (2800–3000 cm^–1^ for C–H stretches or 1700–1750 cm^–1^ for C=O stretches) confirms the effectiveness of the purification protocol in removing residual organic contaminants from the nanocomposite system.

Figure 2B displays the ultraviolet–visible (UV–vis) extinction spectrum of the AgAu/SiO_2_. UV–vis spectroscopy provides valuable insights into electronic transitions and plasmonic behavior across the spectral range of 250–1000 nm. The measured spectrum exhibits two well-defined absorption features, providing fundamental insights into the material’s optical properties. The first distinct absorption peak appears at 288 nm, originating from intrinsic electronic transitions within the SiO_2_ matrix. This feature specifically corresponds to excitonic absorption associated with oxygen vacancy defects or surface states present in the silica framework. The second absorption manifests as a broad band extending from 450 to 650 nm, with a maximum intensity at 550 nm, which is attributed to the localized surface plasmon resonance (LSPR) phenomenon occurring in the AgAu nanoshells, resulting from the collective oscillation of conduction electrons within the bimetallic nanostructure.

The observed LSPR position at 550 nm represents an intermediate value between the characteristic plasmon resonances of pure silver nanoparticles (∼400 nm) and gold nanoparticles (∼520 nm). This spectral behavior provides strong evidence for the formation of an Ag–Au alloy phase, as opposed to phase-segregated metallic domains. The alloy formation leads to modified dielectric properties and electronic structure that account for the observed plasmonic behavior.

After successfully preparing the AgAu NSs/SiO_2_ material, XPS analyses were performed to evaluate the material’s chemical composition and oxidation states, assessing the electronic behavior of the AgAu NSs within the silica matrix (Figure 3). Thus, the high-resolution XPS spectrum for Ag, displayed in Figure 3A, shows binding energies (BE) for two different oxidation states of silver. First, the metallic silver (Ag^0^) is characterized by BEs at 367.1 eV for Ag 3d_5/2_ and 373.1 eV for Ag 3d_3/2_. Additionally, the oxidized silver species (Ag^+^) exhibit BEs at 367.7 eV for Ag 3d_5/2_ and 373.7 eV for Ag 3d_3/2_.^39^ Interestingly, the technique revealed that the quantities of Ag^+^ and Ag^0^ were nearly the same. As depicted in Figure 3B, the peaks identified at BE of 83.5 and 87.2 eV are attributed to metallic gold (Au^0^) species related to the Au 4f_5/2_ and Au 4f_7/2_ spin–orbital splitting, respectively.^49^ In contrast to the analysis of Ag species, the evaluation revealed predominantly reduced Au species, although oxidized species can be present in minimal quantities.

**Figure 3:**
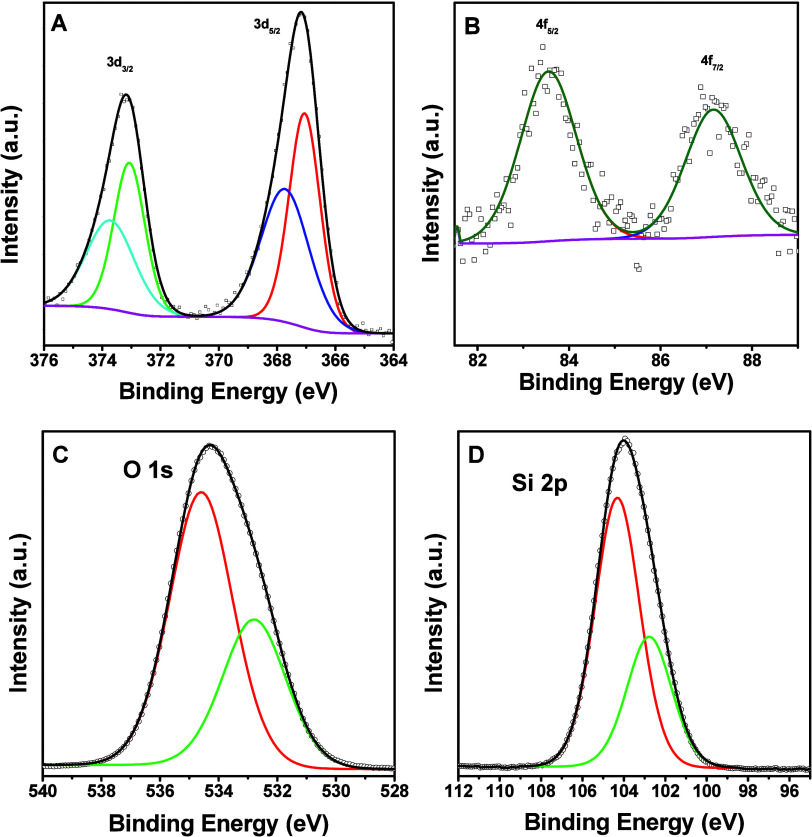
High-resolution XPS spectra of (A) Ag, (B) Au, (C) O, and (D) Si.

In the high-resolution O 1s spectrum (Figure 3C), the peak centered at 532.8 eV is attributed to oxygen species chemically bound to the surface, such as hydroxyl groups (−OH) or Si–O–M linkages at the interface between the Ag–Au nanoshells and the SiO_2_ support, in agreement with literature for metal–oxide systems. On the other hand, the peak located at 534.4 eV corresponds to physisorbed molecular water, a well-documented feature for nanostructured materials and oxide surfaces exposed to ambient conditions, where water adsorption typically manifests.^50^ Quantitative peak deconvolution indicates that the physisorbed water accounts for 65% of the total O 1s signal, while the contribution from surface hydroxyls and Si–O–M species represents the remaining 35%.

The high-resolution Si 2p XPS spectrum displays two distinct components located at 102.8 and 104.3 eV (Figure 3D). The peak at 102.8 eV is attributed to Si–O bonds in substoichiometric silicon oxides (Si^*n*+^, where *n* < 4), typically associated with partially oxidized silicon species at the interface. In contrast, the peak at 104.3 eV corresponds to fully oxidized silicon (Si^4+^), characteristic of stoichiometric SiO_2_. Quantitative analysis reveals that the suboxide component accounts for 32.7% of the total Si 2p signal, whereas the SiO_2_ contribution represents 67.3%.^51,52^

### Materials Electrochemical Characterizations

In the explored study, the CV technique was employed to understand the electrochemical properties of several modified GCEs, as showcased in Figure 4A. The experimental configuration comprised a bare GCE, a GCE modified with a 5.00% Nafion solution (NF/GCE) to investigate the impact of its ionic conductivity and stability, a GCE modified with silver–gold nanostructures coupled with silica (AgAu NSs/SiO_2_/GCE) to leverage their enhanced electrocatalytic activity through superior surface area and electron transport, and finally, a GCE enhanced with both AgAu NSs/SiO_2_ and Nafion (AgAu NSs/SiO_2_/NF/GCE) aiming to synergize their electrochemical benefits. Conducted in a pH 4.00 BR buffer with a potential scan from −0.30 to +1.40 V against an Ag/AgCl reference electrode, the study identified capacitive currents across all materials, indicative of charge accumulation at the electrode–electrolyte interface.

**Figure 4:**
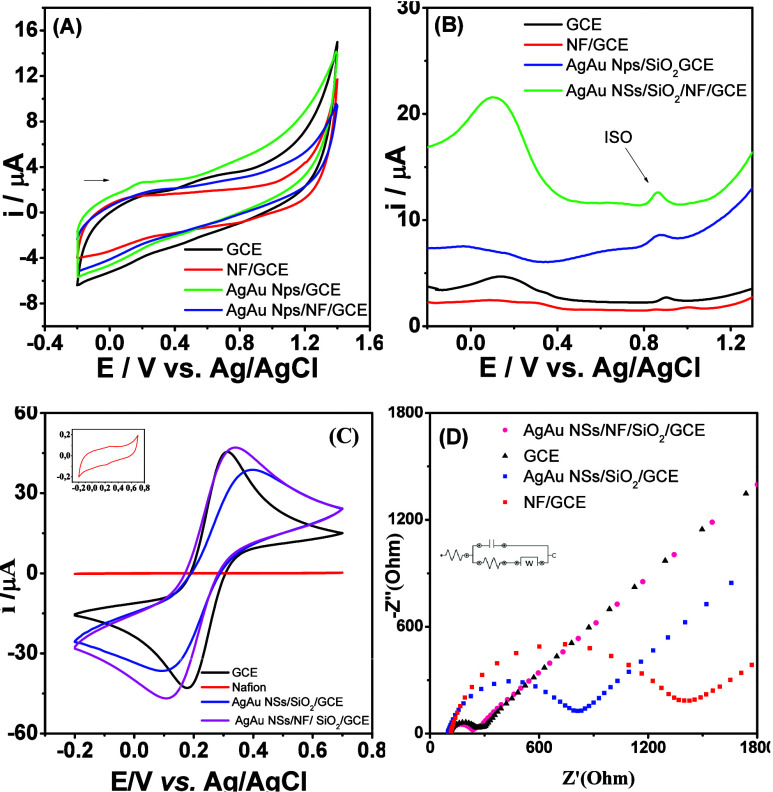
(A) CV profile of GCE; Nafion 5.00% (NF/GCE); AgAu NSs/SiO_2_/GCE; and AgAu NSs/SiO_2_/NF/GCE in 0.1 mol L^–1^ BR pH 4.00 in the presence of 1.0 mg L^–1^ ISO. (B) DPV performed in GCE; Nafion 5.00% (NF/GCE); AgAu NSs/SiO_2_/GCE; and AgAu NSs/SiO_2_/NF/GCE; (C) CV profile of GCE; Nafion 5.00% (NF/GCE); AgAu NSs/SiO_2_GCE; AgAu NSs/NF/SiO_2_GCE; (D) Nyquist plots for GCE; Nafion 5.00% (NF/GCE); AgAu NSs/SiO_2_GCE; AgAu NSs/NF/SiO_2_GCE (Measurements carried out in 0.5 mol L^–1^ KCl solution containing 5.0 mmol L^–1^ ferri-/ferro-cyanide, *n* = 3 for all measurements).

Notably, an electrochemical process at approximately +1.0 V was observed in the Nafion-modified setup, which was absent in the AgAu NSs/SiO_2_/NF/GCE configuration. This observation suggests that the AgAu NSs/SiO_2_ significantly influence Nafion’s electrochemical response. The incorporation of AgAu NSs enhances ionic conductivity and electron transfer by creating pathways within the Nafion matrix, facilitating better proton transport to the electrode surface during redox reactions. Furthermore, the enhanced catalytic activity of the AgAu nanostructures lowers the activation energy for oxidation of water, promoting O_2_ evolution and improving reaction kinetics.

The inclusion of AgAu NSs also modulated the oxygen evolution behavior and slightly reduced capacitive currents at the GCE, indicating an optimized electrochemical interface. This nuanced investigation elucidates the significant role that each material composition and surface modification can play in tailoring the electrochemical performance of the electrodes.

Adding to the CV data depicted in Figure 4C, EIS was employed to elucidate the interfacial electron transfer dynamics and electrical properties of the electrode/solution interface.^53,54^ The impedance data were fitted to a carefully designed equivalent circuit model, illustrated in Figure 4D, to quantitatively interpret the charge transfer resistance (*R*_ct_) associated with the [Fe­(CN)_6_]^3–^/[Fe­(CN)_6_]^4–^ redox system. By determining the semicircular region of the Nyquist plot, we measured Rct for unmodified GCE (155.05 Ω), Nafion/GCE (1.26 kΩ), AgAu NSs/SiO_2_/GCE (722.68 Ω), and AgAu NSs/NF/SiO2/GCE (129.96 Ω).^55–57^

According to the results presented, we can observe a reduction in the final electrode AgAu NSs/NF/SiO_2_/GCE. This reduction, although not drastically low, shows the enhanced electron transfer capabilities imparted by the nanostructured AgAu nanoshells. Ag nanostructures typically possess a high surface-to-volume ratio and superior metallic conductivity, which likely facilitates more effective electrocatalytic activity. Additionally, the Au nanoparticles exhibit electrocatalytic properties. This synergy is evident in the overall improvement in conductivity observed for the redox probe.

### ISO Selective Oxidation on AgAu NSs/SiO_2_/NF/GCE

Differential pulse voltammetry analysis (Figure 4B) demonstrates how AgAu NSs/SiO_2_/Nafion modifications fundamentally alter the oxidation behavior of ISO. Compared to the unmodified GCE, the composite electrode exhibits both a 4 mV reduction in oxidation potential and a 220% increase in peak current – two key metrics that confirm enhanced electrocatalytic activity. Such simultaneous improvements in thermodynamic favorability and kinetic efficiency suggest the bimetallic nanoshells actively participate in the charge transfer process rather than simply increasing surface area.

Optimal performance emerges from careful control of modification parameters. Systematic evaluation of suspension volumes (3–15 μL, Figure S1) reveals that 10 μL provides ideal nanomaterial coverage, balancing three critical factors: (1) complete active site accessibility, (2) minimized diffusion limitations, and (3) maintained electrical connectivity throughout the film. This optimized architecture achieves optimal surface utilization while preventing the conductivity losses observed at higher loadings.

Current enhancement stems from synergistic interactions between components. Nafion’s ion-conducting domains likely orient ISO molecules near catalytically active Au sites, while the SiO_2_ support prevents nanoshell aggregation. Such molecular-scale cooperation explains why the composite outperforms either modification alone, establishing a design strategy that is transferable to other electrochemical sensing platforms.

### Scan Rate and pH Influences

A comprehensive investigation was conducted across a pH spectrum ranging from 2.0 to 10.0 to assess the oxidation behavior of ISO under varying H^+^ concentrations, as detailed in Figure S2-A. pH measurements were acquired using an AgAu NSs/SiO_2_/NF/GC electrode in a 0.10 mol L^–1^ BR supporting electrolyte. Figure S2-A revealed a maximum peak current at a pH of 3.0, after which a decrease in signal intensity and broadening of the peak base were noted. This indicates that pH 3.0 is the optimal condition for ISO detection. The data presented in Figure S2-B elucidate a pronounced correlation between peak potentials (*E*_p_), peak current (*I*_p_), and the pH value.

Such pH effect is attributed to the availability of H^+^ ions in the bulk, which directly influences the kinetics of the oxidation process of ISO. At pH 3.0, the concentration of protons appears to be optimal for facilitating the electron transfer associated with ISO oxidation. As the pH increases, the availability of H^+^ ions decreases, resulting in reduced signal intensity and broader peak shapes. We suggest that at higher pH values, the diminished proton concentration limits the efficiency of the oxidation process, resulting in a less defined signal and lower peak currents.

Additionally, the shift in peak potentials with varying pH values indicates the dependence of the oxidation process on H^+^ concentration. However, since the redox process is not reversible, the observed behavior does not follow the expected linear relationship described by the Nernst equation. Thus, while we can suggest that an electrochemical process involving protons and electrons occurs, the exact mechanism is more complex than a straightforward proton–electron transfer in equilibrium.

Subsequently, the effect of varying scan rates on the CV response was examined using BR at a concentration of 0.10 mol L^–1^, with ISO added to achieve a final concentration of 0.20 mg L^–1^, as shown in Figure S2-C. The cyclic voltammograms were recorded over scan rates ranging from 10 to 100 mV s^–1^. Peak current values were graphically plotted against the scan rate in Figure S2-D. Figure S2-E demonstrates a linear relationship between the peak current (*I*_p_) and the square root of the scan rate (*v* ^1/2^), indicating that the system’s behavior is predominantly governed by diffusion, as evidenced by the coefficient of determination (*R* ^2^ = 0.9993). Moreover, the relationship between the peak current’s logarithm and the scan rate’s logarithm, illustrated in Figure S2-F, yielded a slope of 0.71. This value is statistically closer to the theoretical slope of 0.5, characteristic of a diffusion-controlled process, rather than 1.0, which is indicative of an adsorption-controlled mechanism. In light of this result, the half-height width (*W*_1/2_) at lower scan rates was utilized to estimate the number of electrons involved in the ISO oxidation process, as depicted in the voltammogram of Figure S2-G. Given that the *W*_1/2_ value of the anodic peak is 0.067 V, the oxidation mechanism in this context implicates the transfer of 2 electrons. Evidence in the literature suggests that 2 protons are involved in this reaction,^58^ as further detailed in Figure S2-H. This finding underscores the electrochemical nature of the ISO oxidation process under the examined conditions.

### Optimizations toward ISO Detection

Choosing the proper voltammetric method is crucial for developing an electrochemical sensor, especially for enhancing its sensitivity toward the oxidation of ISO. In a comparative study involving LSV, DPV, and SWV, the parameter settings of each technique were carefully equalized to ensure a fair assessment. The study focused on detecting 0.15 mg L^–1^ of ISO in a 0.10 M Britton-Robinson (BR) buffer solution at pH 3.00 using an AgAu NSs/SiO_2_/NF/GCE platform. Results, showcased in Figure S3, revealed DPV as the more suitable technique due to its higher peak current values. Therefore, DPV was selected for the sensor development due to its heightened sensitivity in this application.

Following the selection of DPV as the preferred technique, further experiments were conducted to fine-tune its parameters to enhance the experimental setup’s efficiency. Parameters such as the scan rate, pulse duration (*t*_pulse_), and pulse amplitude (*E*_amplitude_) were varied to gauge their impact on the sensor’s current response. The scan rate was tested over a range from 10 to 50 mVs^–1^, *t*_pulse_ from 0.005 to 0.02 s, and *E*_amplitude_ from 10 to 100 mV, as detailed in Figure S4 and Table S1. We identified the most effective settings for DPV measurements to be a scan rate of 30 mV/s, a *t*_pulse_ of 0.008 s, and an *E*_amplitud_ of 70 mV. These optimized DPV parameters were then applied in further experiments to ensure the electrochemical sensor’s effectiveness in detecting ISO.

In addition, the effectiveness of various supporting electrolytes was evaluated through DPV, identifying the BR supporting electrolyte at a concentration of 0.10 M as the optimal choice for detecting ISO. Selected from a group that included Mcllvaine, BR, PBS, and Citrate buffers, based on their pH buffering capacities (that must include the pH 3.00), the BR buffer demonstrated superior performance by producing a consistent and pronounced peak current within the 0.80 to 1.00 V range, with minimal measurement deviations, as detailed in Figure S5. Further optimization trials, assessing concentrations from 0.025 to 0.25 mol L^–1^, corroborated the selection of 0.10 mol L^–1^ as the concentration that significantly enhanced peak current signals (Figure S5-C), marking it as the most effective for subsequent experiments.

### Determination of ISO

Following optimization, the analytical performance of the AgAu NSs/SiO_2_-based sensor was thoroughly evaluated. Figure 5 shows a calibration curve plotted with ISO concentration values ranging from 0.75 to 40.0 μg L^–1^. These experiments were conducted under rigorous circumstances, with a pH of 3.00 and a supporting electrolyte content of 0.10 mol L^–1^ BR, and each test was performed three times. Non-Faradaic peak current values were removed by subtracting the baseline, leaving just the currents directly connected to ISO oxidation. The resultant calibration curve (Figure 5B) demonstrated a clear linear response (0.75–20.0 μg L^–1^) that could be accurately described by the equation *I*_p_ = 0.23­[ISO] – 0.08, with an *R* ^2^ value of 0.9930, indicating a significant correlation between current and ISO concentration. It is crucial to remember that the Maximum Residue Limit (MRL) for ISO differs by jurisdiction.^59,60^ The detection limit (LOD) was set at 0.011 μg L^–1^, indicating the lowest ISO concentration that can be reliably detected. The quantification limit (LOQ) was set at 0.037 μg L^–1^, indicating the lowest ISO level that can be accurately quantified. The sensor’s sensitivity was defined by the slope of the calibration curve, which was 0.23 μA L μg^–1^. The values of LOD and sensitivity enable the sensor to efficiently perform analyses in accordance with EU requirements, indicating that it is well-suited for detecting ISO in simulated blood plasma and food extracts, such as tomatoes. These findings demonstrate that the sensor can be utilized in real-world studies while meeting stringent regulatory criteria that would be impossible to achieve without deliberate surface modification of the GCE.

**Figure 5:**
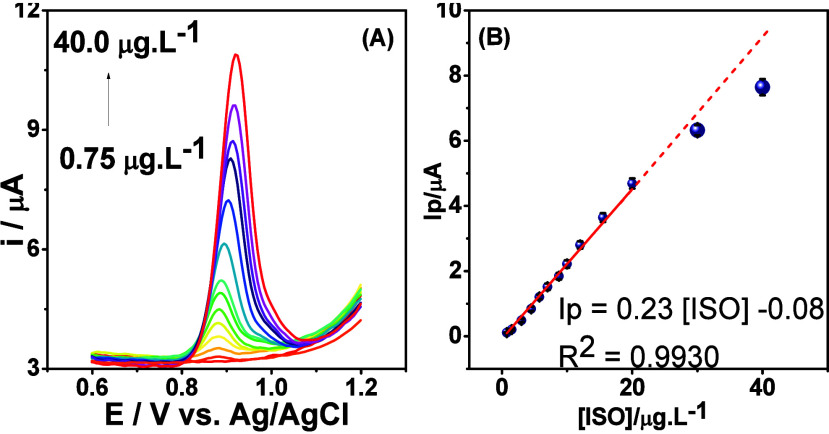
(A) DP voltammograms for ISO in a concentration ranging from 1.00 to 40.00 μg L^–1^. (B) Corresponding calibration curve (*n* = 3 for all measurements).

### Reproducibility (Interday), Repeatability (Intraday), Selectivity, and Stability of the AgAu NSs/SiO_2_/NF/GCE Probe

Evaluating the AgAu NSs/NF/GCE sensor’s efficacy revealed its exceptional reliability and precision, with a repeatability of 3.28%, based on five consecutive tests using a single electrode premodified five times and an ISO concentration of 5.35 μg L^–1^, as shown in Figure S6-C. The sensor also exhibited remarkable reproducibility, with a relative standard deviation (RSD) of 1.10% in five repeated measurements using the same setup, as shown in Figure S6-A. These findings demonstrate the sensor’s robustness and capability to produce consistent and uniform results, a crucial aspect for applications demanding high precision, such as medical diagnostics or environmental monitoring.^61^

The method’s sensitivity was investigated by routinely testing compounds found in both ground and surface waters. Furthermore, an examination incorporated acids and heavy metals that are often found in human blood and food. DPV measurements were performed with 1.50 μg L^–1^ of ISO to assess this in the presence of various interferents. The obtained voltammograms are shown in Figures S6-E–H. A proportion of 1:5 was used in the electrochemical cell. Results in Table S2 demonstrated that the effect of the evaluated organic pollutants was less than 5.00% on the peak current. Uric acid (+14.02) and pirimicarb (+11.21) showed significant interference. Nonetheless, the deviation in current observed in the presence of these compounds remained below 15%, indicating good sensor sensitivity.^46^

The sensor’s stability was evaluated over a period of 7 weeks. Throughout this time, the dispersion was kept refrigerated at 7 °C but was used at room temperature to fabricate the film on the GCE. The current responses generated by AgAu NSs/SiO_2_/NF/GCE, using the same dispersion and applying DPV under optimized conditions for ISO, decreased by approximately 5.67% after 7 weeks compared to the initial response. These results indicate that the AgPt NTs/NF/GCE maintained stability for electrochemical applications during this period.

### Real-Sample Application

Using the standard addition technique, research was conducted to assess the accuracy of ISO determination in aquaculture water, simulated plasma, and tomato extract (Figure S7). Using simulated plasma samples in research and clinical applications can be an effective and practical alternative to real plasma.^62^ Simulated plasma is cost-effective, ethically sound, and consistently reproducible, providing a reliable foundation for experiments.^62,63^ Unlike real plasma, which varies between donors, simulated plasma ensures uniformity, reducing variability and enhancing the accuracy of results. It poses lower biohazard risks, making it safer to handle. To ensure reproducibility, each spiking sample was examined three times for each concentration level (*n* = 3). The analysis demonstrated negligible matrix interference, as evidenced by the consistency in slope between the standard addition technique within the matrix and the calibration curve. This indicates that other substances in the samples have no significant impact on the accuracy of the ISO measurement. Accurate quantification of ISO was demonstrated by the recovery values obtained, which varied from 83.0 to 100.4% (Table S3). Notably, recovery percentages in this range indicate that the measured concentrations closely correspond statistically to the predicted amounts.^46^ Overall, the study demonstrates the method’s robustness and suitability for accurately determining ISO concentrations in various samples, which is crucial for applications such as aquaculture water quality monitoring, human plasma analysis, and food safety assessment.

The Analytical GREEnness Metric Approach (AGREE) was employed further to evaluate the environmental friendliness of the suggested method. AGREE helps assess a method’s analytical performance and environmental effect, which is critical. The proposed approach is an excellent alternative to ISO determination in water samples, as it is sustainable and beneficial to the environment, as demonstrated by the AGREE figure of merit assessment of 0.89 in Figure S8.

A variety of modified electrodes used for detecting ISO is shown in Table 1. We suggested a technique that demonstrated exceptional accuracy in identifying ISO, outperforming findings reported in respected scientific literature. This presents a low detection limit, allowing for the accurate and precise identification of even minimal ISO concentrations in various sample matrices, as well as a linear operating range within the usual concentration range. The presented sensor stands out due to its simple manufacturing technique, as opposed to approaches that employ graphene, multiwalled carbon nanotubes, and molecularly imprinted polymers. It is simple to carry out and straightforward to fabricate.

**Table 1:** Analytical Performance of Different Electrochemical Methods for ISO Determination

modification/working electrode	method	linear range (μg L^–1^)	LOD (μg L^–1^)	LOQ (μg L^–1^)	pH	sensitivity (nA L μg^–1^)	ref
^a^PtNPS/CS/GCE	DPAdSV	40–1000	7.0	2.0	2.0	0.81	^64^
^b^graphene/SPE	SWV	2.0–100	20.0	2.0	2.0	0.43	^65^
^c^GO-MWCNTsCOOH/GCE	SWV	62–3094	21.0	6.5	6.5	1.48	^1^
^d^NiAl-LDH/CPE	SWV	4.12–37.13	0.21	4.5	4.5	0.68	^66^
^e^Nafion/GCE	SWV	18.5–4126.0	6.20	1.0	1.0	2.88	^67^
^f^NiO/V2O5/rGO/GCE	DPV	1.85–6188.6	1.03	4.0	4.0	0.48 × 10^–6^	^58^
AB/GCE^g^	DPV	103.15–4126	19.8		3.0	0.28	^68^
MIP-GCE^h^	SWV	0.51–206.3	0.5	1.9		2.40	^69^
(CG-NTC-CuO)-CPE^i^	CV	0.3–200	0.1	0.3			^70^
AgAu NSs/SiO_2_/NF/GCE^j^	DPV	0.75–20.0	0.011	0.037	3.0	230	this work

a Platinum nanoparticles/chitosan supported on a glassy carbon electrode.

b Graphene screen-printed electrode.

c Graphene oxide–modified multiwalled carbon nanotubes on a glassy carbon electrode.

d NiAl layered double hydroxide on a carbon paste electrode;

e Nafion supported on a glassy carbon electrode.

f Nickel oxide/vanadium oxide/reduced graphene oxide supported on a glassy carbon electrode.

g Acetylene black nanoparticles modified glassy carbon electrode.

h Molecularly imprinted polymer-modified glassy carbon electrode.

i Carbon paste electrode incorporated with carbon nanotubes and synthesized copper oxide nanoparticles.

j Silver nanotubes with gold nanoparticles supported on SiO_2_ on a glassy carbon electrode.

Furthermore, the approach reduces the need for organic solvents, which improves both the economic and environmental impact of its use. It saves time during experimental setup since the one-step adjustment approach is quick. Additionally, in the case of biofluids like human plasma, where it is preferable to use the smallest possible sample volume, this feature is particularly beneficial. These distinguishing qualities make the sensor created in this work particularly advantageous for detecting ISO compared to previous approaches.

## Conclusions

In this work, we successfully demonstrated that hollow AgAu nanoshells supported on SiO_2_ represent an optimal design for electrochemical ISO detection, achieving an exceptional detection limit of 0.011 μg L^–1^ – over 10 times more sensitive than most reported electrochemical sensors. The key innovation lay in the synergistic combination of the bimetallic hollow nanostructure and SiO_2_ support, which collectively enhances electron transfer while preventing nanoparticle aggregation. First, its 220% higher current response compared to conventional electrodes directly translates to superior sensitivity for trace-level detection. Additionally, the one-step modification process using Nafion is more straightforward and cost-effective than existing methods that rely on graphene, molecular imprinting, or complex nanocomposites. Furthermore, it maintains this high performance across real-world matrices, including aquaculture water, food extracts, and biological fluids, where other sensors typically fail due to matrix interference.

With a wide linear range (0.75–20.0 μg L^–1^), rapid response, and excellent reproducibility (<3.3% RSD), this platform offers a practical solution for monitoring ISO residues in environmental, agricultural, and clinical settings. The work establishes a new benchmark for pesticide sensors by proving that nanomaterial design, rather than complex modifications, can yield unmatched sensitivity and robustness.

## Environmental Significance Statement

We present a novel AgAu NSs/SiO_2_/NF/GCE electrochemical sensor with significant promise for elucidating environmental processes and mitigating associated impacts on food safety, ecological risks, and human health in detecting Isoproturon (ISO), 3-(4-isopropylphenyl)-1,1-dimethylurea. ISO demands extreme urgency for sensitive detection methods, such as the one we presented. This sensor’s ability to detect ISO across diverse matrices, including aquaculture water, tomato extract, and human plasma, shows its versatility. The sensor exhibits limits of detection and quantification lower than those reported in many previous studies, minimal interference from matrix constituents, and a broad working linear range. Our work is relevant to environmental applications, food quality, and human health monitoring.

## Supplementary Material



## Notes

Influence of modifier aliquot (3.0–15.0 μL) on ISO response; pH effect, scan rate studies, and electrochemical oxidation mechanism of ISO at AgAu NSs/SiO_2_/GCE; comparison of LSV, SWV, and DPV for ISO detection; optimization of DPV parameters: amplitude, scan rate, and pulse time; effect of supporting electrolyte type and concentration on ISO response; reproducibility and interference studies using different electrodes and analytes; ISO recovery in spiked surface water, tomato extract, and human plasma; greenness evaluation using AGREE method; DPV parameter optimization; comparative interference analysis; and recovery data from standard addition method (PDF)
