# Supplementary material for: Electrocatalytic Hollow AgAu/SiO2 Sensor for Multi-Matrix Isoproturon Monitoring
Source: ACS Omega. 2025 Aug 12;10(33):37474–85. doi: 10.1021/acsomega.5c02480 (PMC12391933; doi:10.1021/acsomega.5c02480)
Supplement: Supplementary file 1 [file ao5c02480_si_001.pdf]

Electrocatalytic Hollow $AgAu/SiO_2$ Sensor for Multi-Matrix Isoproturon Monitoring

Jose Antonio de Oliveira Junior,[1] *Antonio Gomes dos Santos Neto,*[2] *Camila Silva de Sousa,*[2] *Rebeca Yatsuzuka,*[3] *Felipe Anchieta e Silva,*[4] *Thenner Silva Rodrigues,*[4] *Marco Aurélio Suller Garcia,*[4,5] *Cristiane Luisa Jost*,*[2] *Sergio Yesid Gomez Gonzalez*,*[1]

[1]Departamento de Engenharia Química e Engenharia de Alimentos, Universidade Federal de Santa Catarina, Campus Universitário Trindade, 88040-900, Florianópolis, SC, Brazil.

[2]ampere - Laboratório de Plataformas Eletroquímicas, Departamento de Química - Universidade Federal de Santa Catarina, Campus Universitário Trindade, 88040-900, Florianópolis, SC, Brazil.

[3]Central Analítica, Instituto de Química, Universidade de São Paulo, Av. Prof. Lineu Prestes, 748, 05508-000, São Paulo, SP, Brazil.

[4]Programa de Engenharia de Nanotecnologia, Instituto Alberto Luiz Coimbra de Pós-Graduação e Pesquisa em Engenharia, COPPE, Universidade Federal do Rio de Janeiro, Av. Horácio Macedo, 2030, 21941-972, Rio de Janeiro, RJ, Brazil.

[5]Departamento de Química, Universidade Federal do Maranhão, Av. dos Portugueses,1966, 65080-805, São Luis, MA, Brazil.

---

*Corresponding Authors:
Sergio Yesid Gomez Gonzalez: sergio.gomez@ufsc.br
Cristiane Luisa Jost: cristiane.jost@gmail.com

SUPPORTING INFORMATION

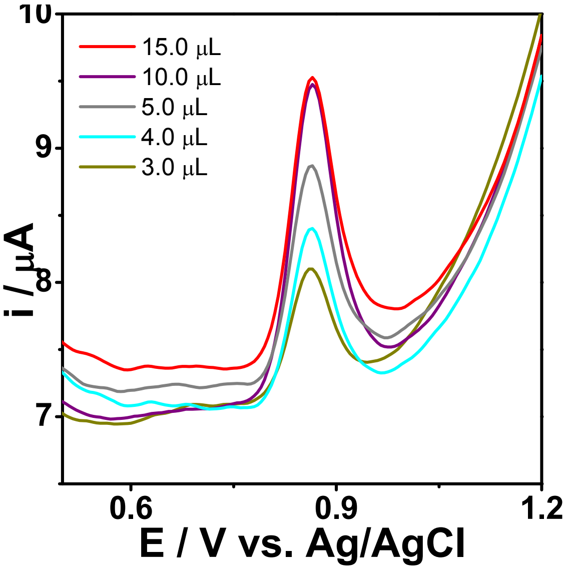

Figure S1 – Figure S1 – Aliquot of modifier, varying from 3.0; 4.0; 5.0; 10.0 to 15.0 µL (conditions: 1.0 mgL^{-1} ISO in BR 0.1 mol L^{-1} and pH 3.00) (*n=3* for all measurements).

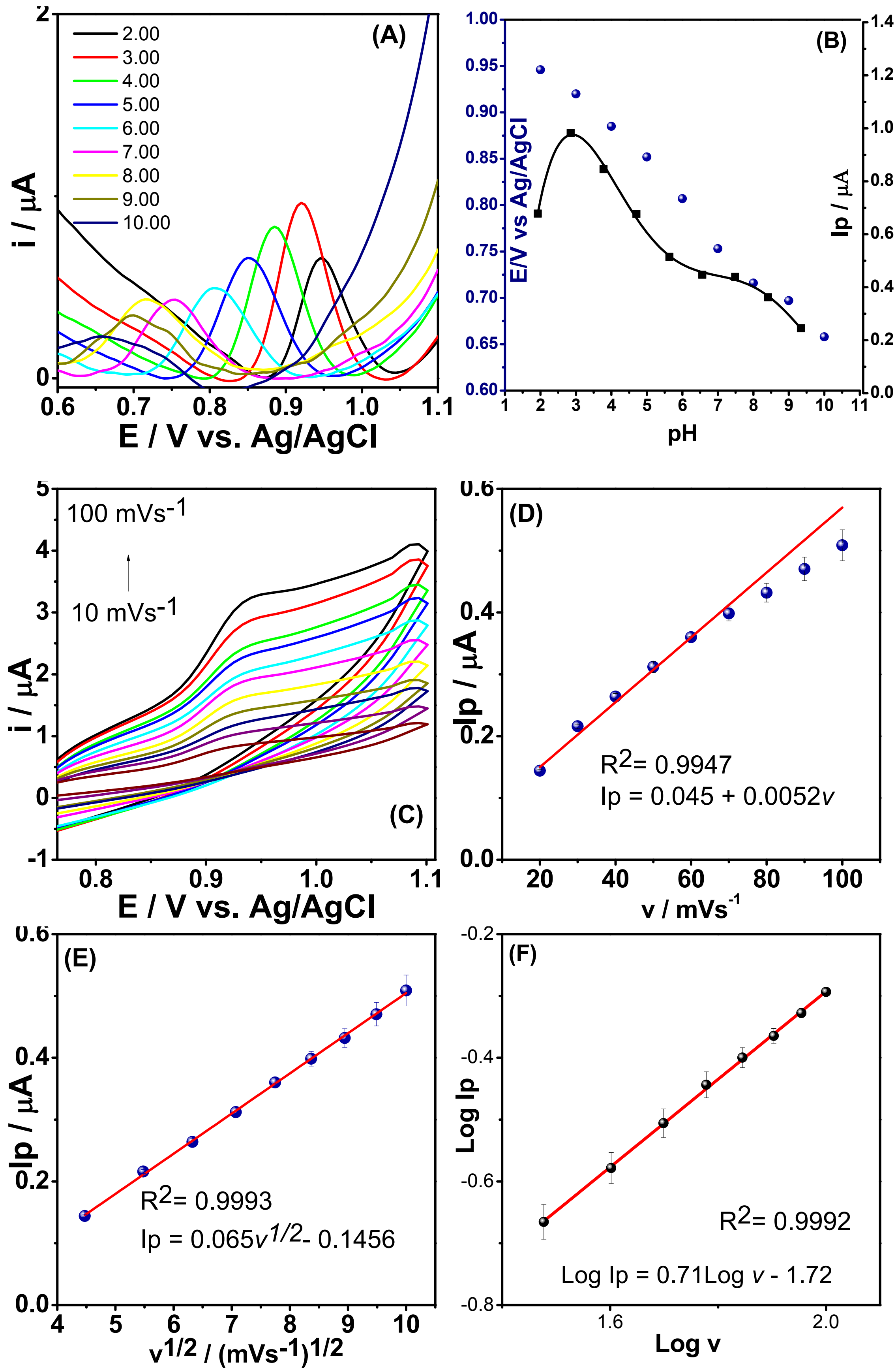

(A)
2.00
3.00
4.00
5.00
6.00
7.00
8.00
9.00
10.00
i / µA
E / V vs. Ag/AgCl
(B)
E/V vs Ag/AgCl
Ip / µA
pH
(C)
100 mVs-1
10 mVs-1
i / µA
E / V vs. Ag/AgCl
(D)
R2= 0.9947
Ip = 0.045 + 0.0052v
oIp / µA
v / mVs-1
(E)
R2= 0.9993
Ip = 0.065v1/2- 0.1456
oIp / µA
v1/2 / (mVs-1)1/2
(F)
R2= 0.9992
Log Ip = 0.71Log v - 1.72
Log Ip
Log v

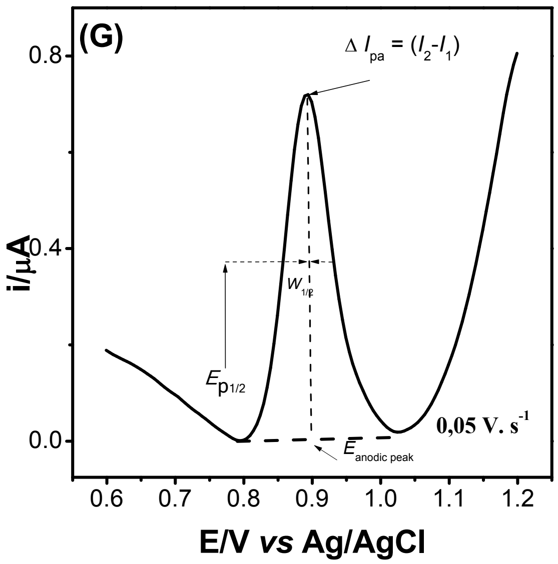

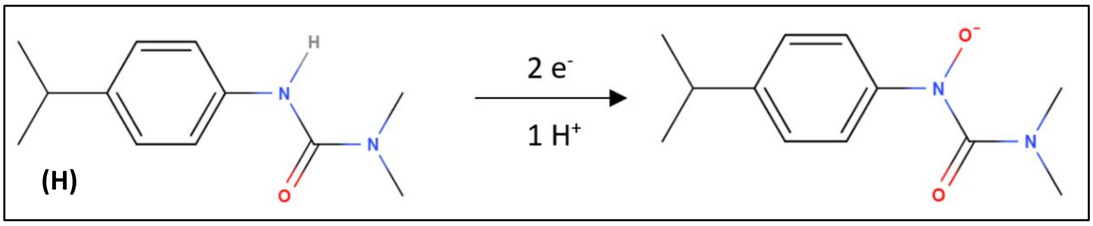

Figure S2 – AgAu NSs/SiO_2/GCE responses: A) DPV in the presence of 0.20 mg L^{-1} ISO in different pHs; B) Anodic peak current and peak potential of ISO at different pHs from 2.0 to 10.0. C) CV at different scan rates from 10 to 100 mV s^{-1}; D) I_p *vs*. scan rate; E) I_p *vs*. square root of scan rate; F) log Ip vs. log scan rate; G) E_p *vs*. log scan rate; H) Electrochemical oxidation proposed mechanism (*n=3* for all measurements).

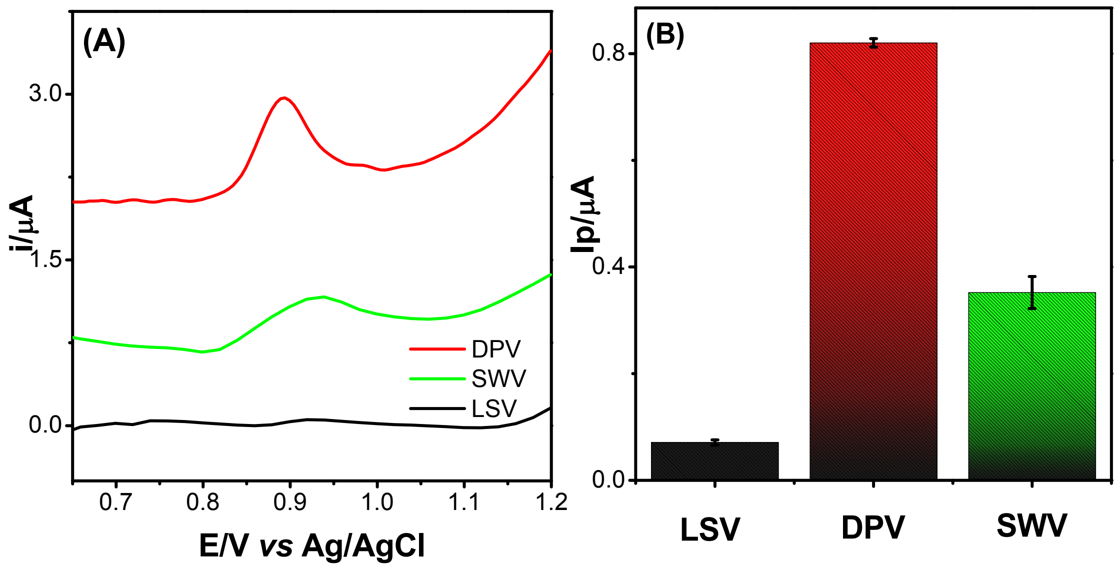

Figure S3 - A) Voltammograms of 0.15 mg L^{-1} ISO by using LSV, SWV, and DPV. B) the corresponding ISO peak current response for LSV, SWV, and DPV (*n=3* for all measurements).

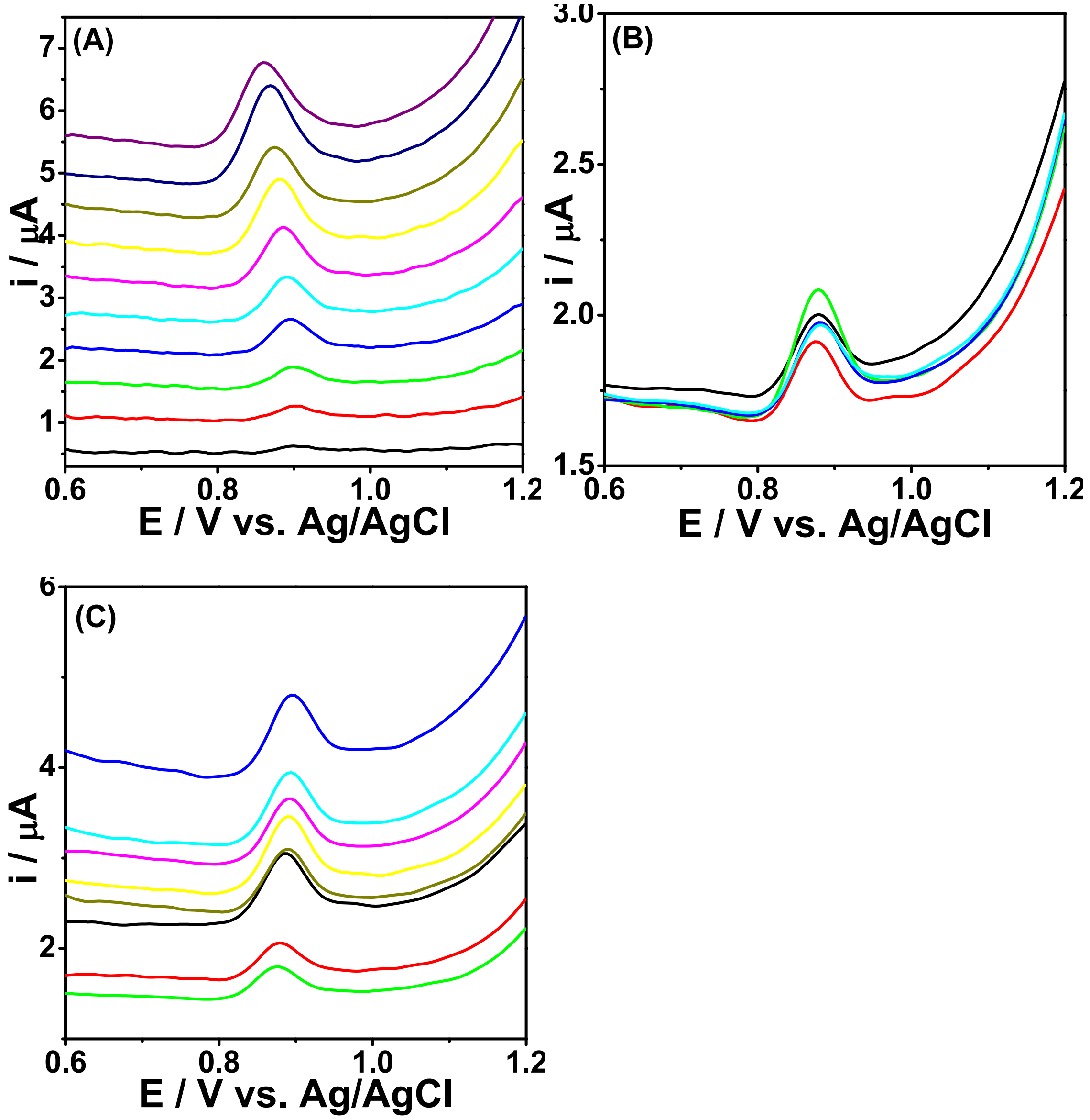

Figure S4 - ISO current response in different DPV conditions. A) Variation of the E amplitude from 0.01 to 0.10 mV. B) Variation of the scan rate from 0.01 to 0.05 mV s^{-1}. C) Variation of the t pulse 0.005 to 0.02 s (*n=3* for all measurements).

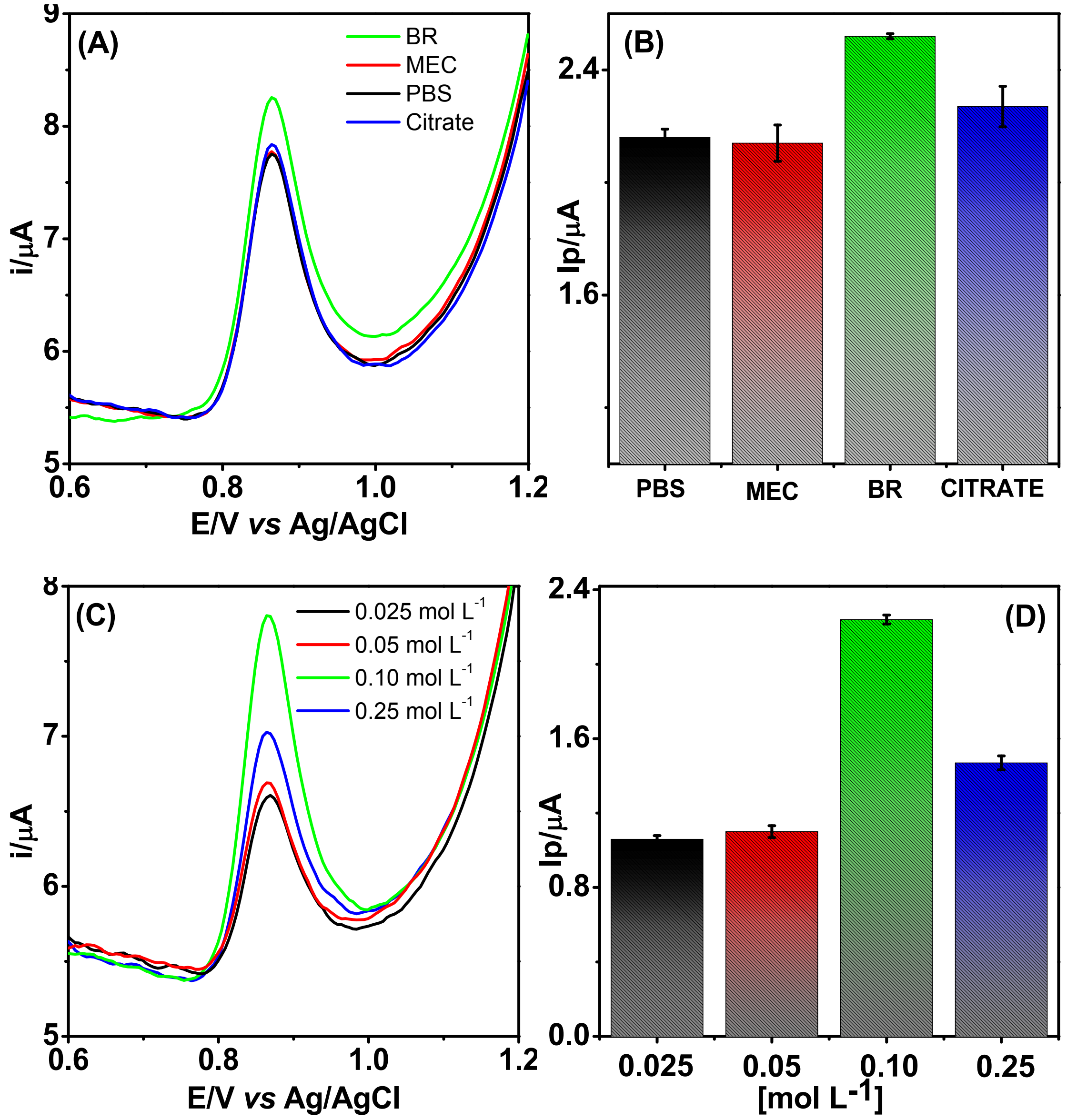

Figure S5 – The choice of supporting electrolyte for ISO response. A) DPV for Britton-Robinson, McIlvainne, PBS, and Citrate 0.10 mol L^{-1}. B) Current responses for different electrolytes. C) Variation of the BR supporting electrolyte concentration from 0.025 to 0.25 mol L^{-1}; and D) the corresponding current responses for different BR supporting electrolyte concentrations (*n=3* for all measurements).

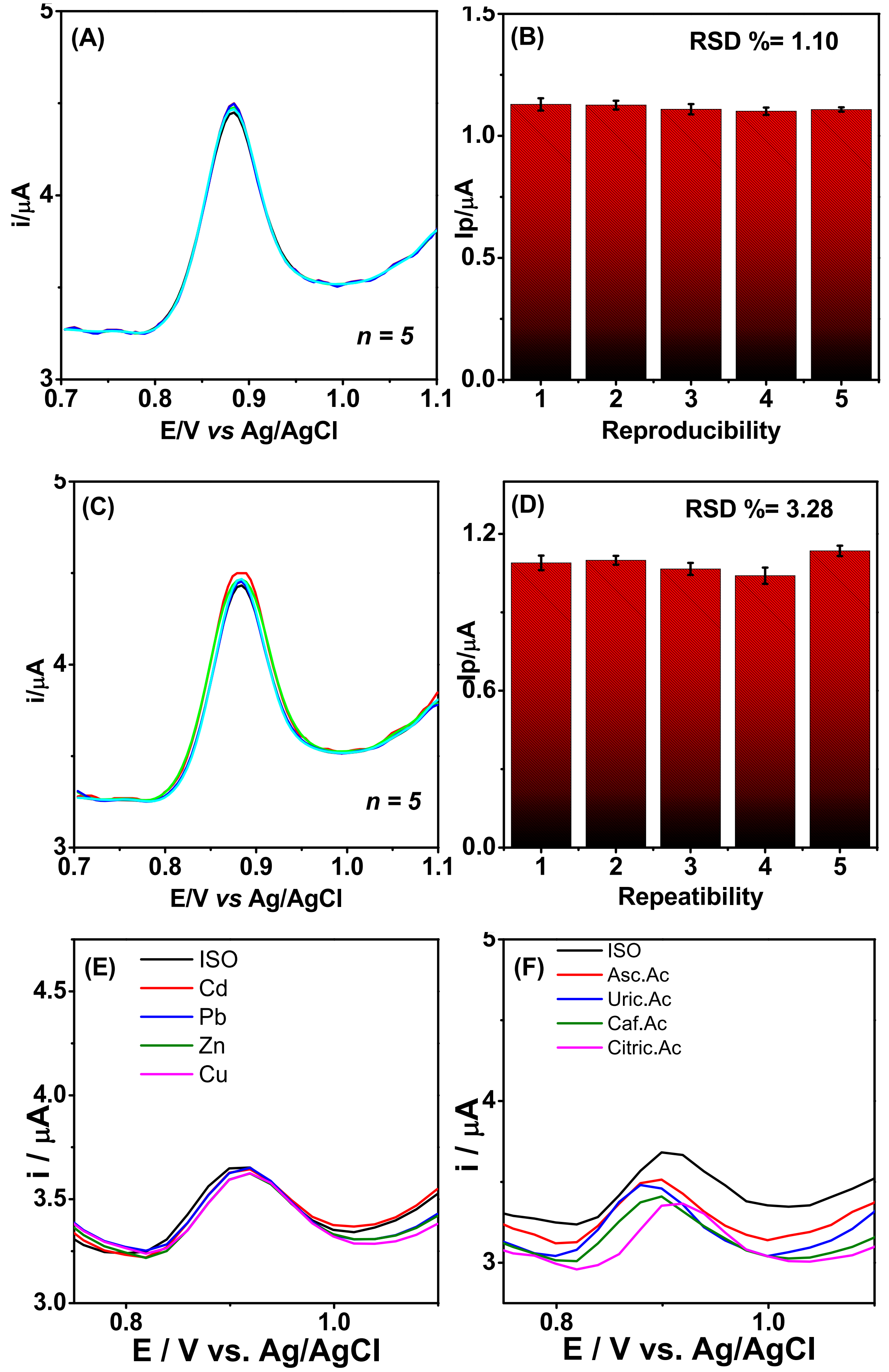

(A)
n = 5
i/μA
E/V vs Ag/AgCl
(B)
RSD %= 1.10
Ip/μA
Reproducibility
(C)
n = 5
i/μA
E/V vs Ag/AgCl
(D)
RSD %= 3.28
Ip/μA
Repeatibility
(E)
ISO
Cd
Pb
Zn
Cu
i / μA
E / V vs. Ag/AgCl
(F)
ISO
Asc.Ac
Uric.Ac
Caf.Ac
Citric.Ac
i / μA
E / V vs. Ag/AgCl

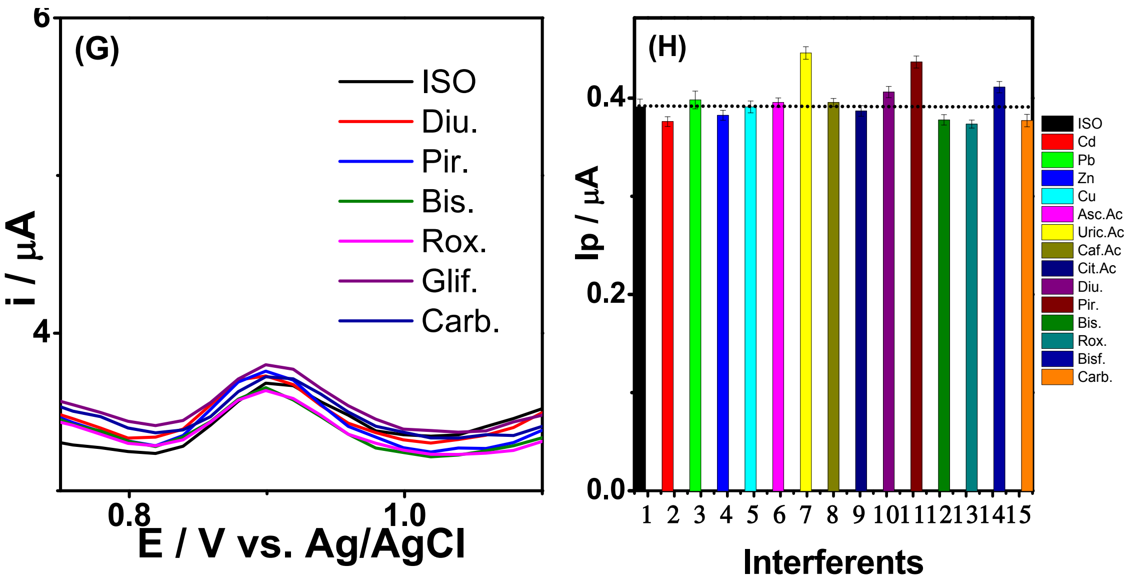

Figure S6 - A) DPV response for ISO at AgAu NSs/SiO_2/GCE using five modified electrodes. B) Peak current response of five different electrodes. C) DPV response for ISO at AgAu NSs/SiO_2/NF/GCE and five measures for the same electrode. D) Peak current response of five different measures for the same electrode. E) Cd, Pb, Zn, and Cu heavy metals interferents (ratio 1:5). F) Ascorbic Acid, Uric Acid, caffeic acid, and Citric Acid interferents (ratio 1:5). G) Diuron, Pirimicarb, Bisphenol, Roxarsone, Gliphosate, and Carbofuran interferents (ratio 1:5) (*n=3* for interferents).

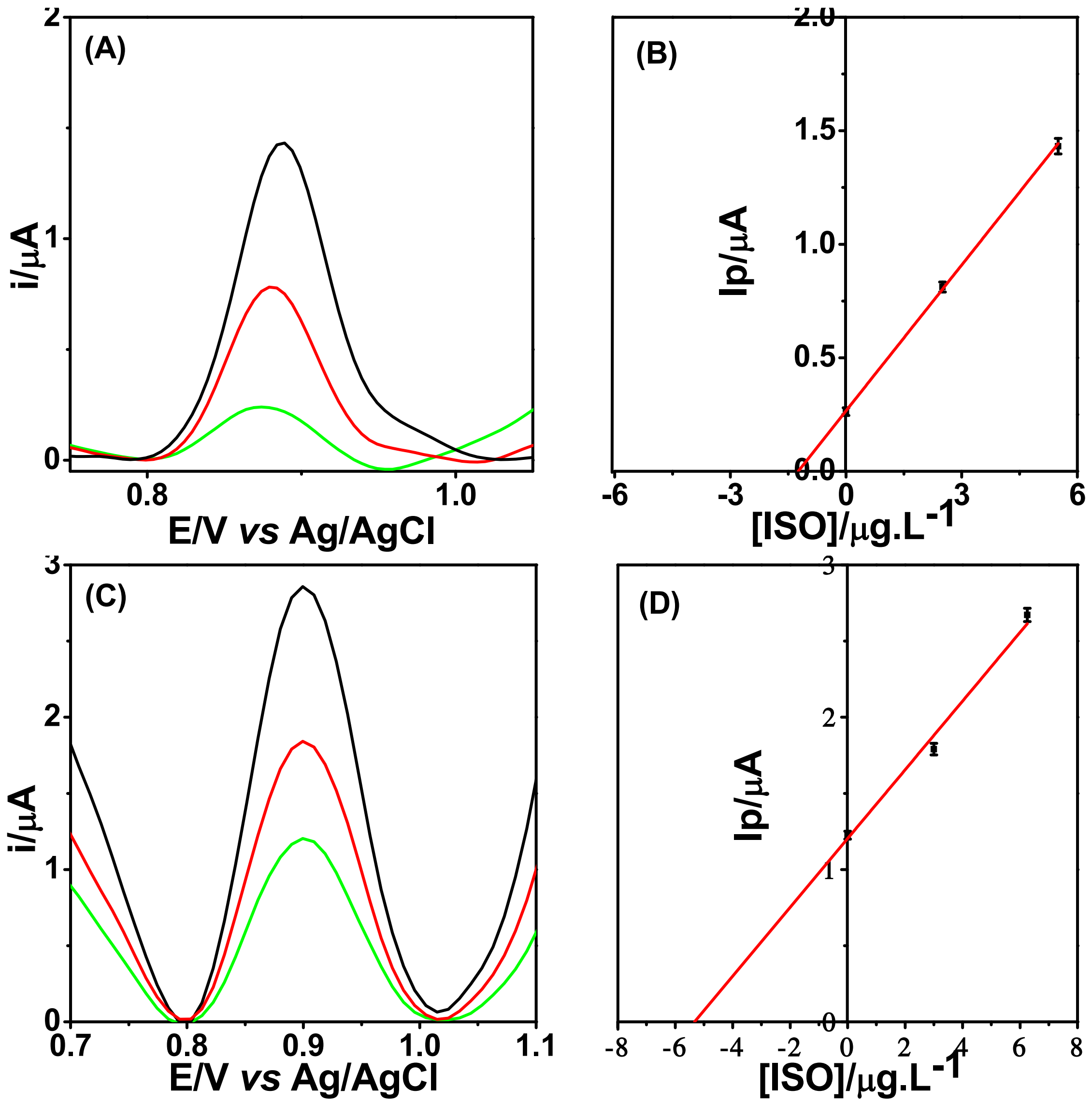

(A)
i/μA
E/V vs Ag/AgCl
(B)
Ip/μA
[ISO]/μg.L-1
(C)
i/μA
E/V vs Ag/AgCl
(D)
Ip/μA
[ISO]/μg.L-1

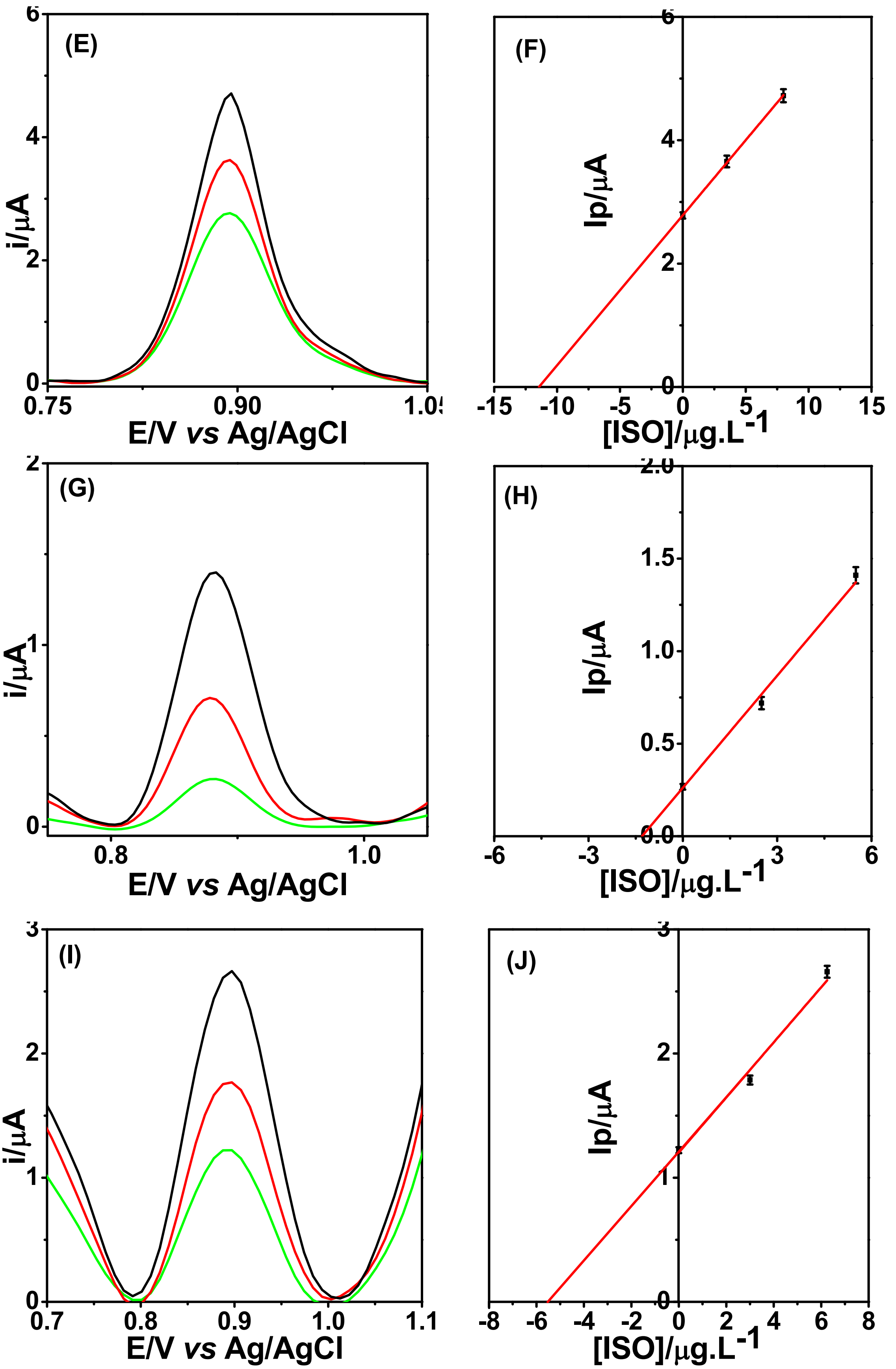

(E)
i/μA
E/V vs Ag/AgCl
0.75
0.90
1.05
0
2
4
6
(F)
Ip/μA
[ISO]/μg.L-1
-15
-10
-5
0
5
10
15
(G)
i/μA
E/V vs Ag/AgCl
0.8
1.0
0
1
2
(H)
Ip/μA
[ISO]/μg.L-1
-6
-3
0
3
6
0.0
0.5
1.0
1.5
2.0
(I)
i/μA
E/V vs Ag/AgCl
0.7
0.8
0.9
1.0
1.1
0
1
2
3
(J)
Ip/μA
[ISO]/μg.L-1
-8
-6
-4
-2
0
2
4
6
8

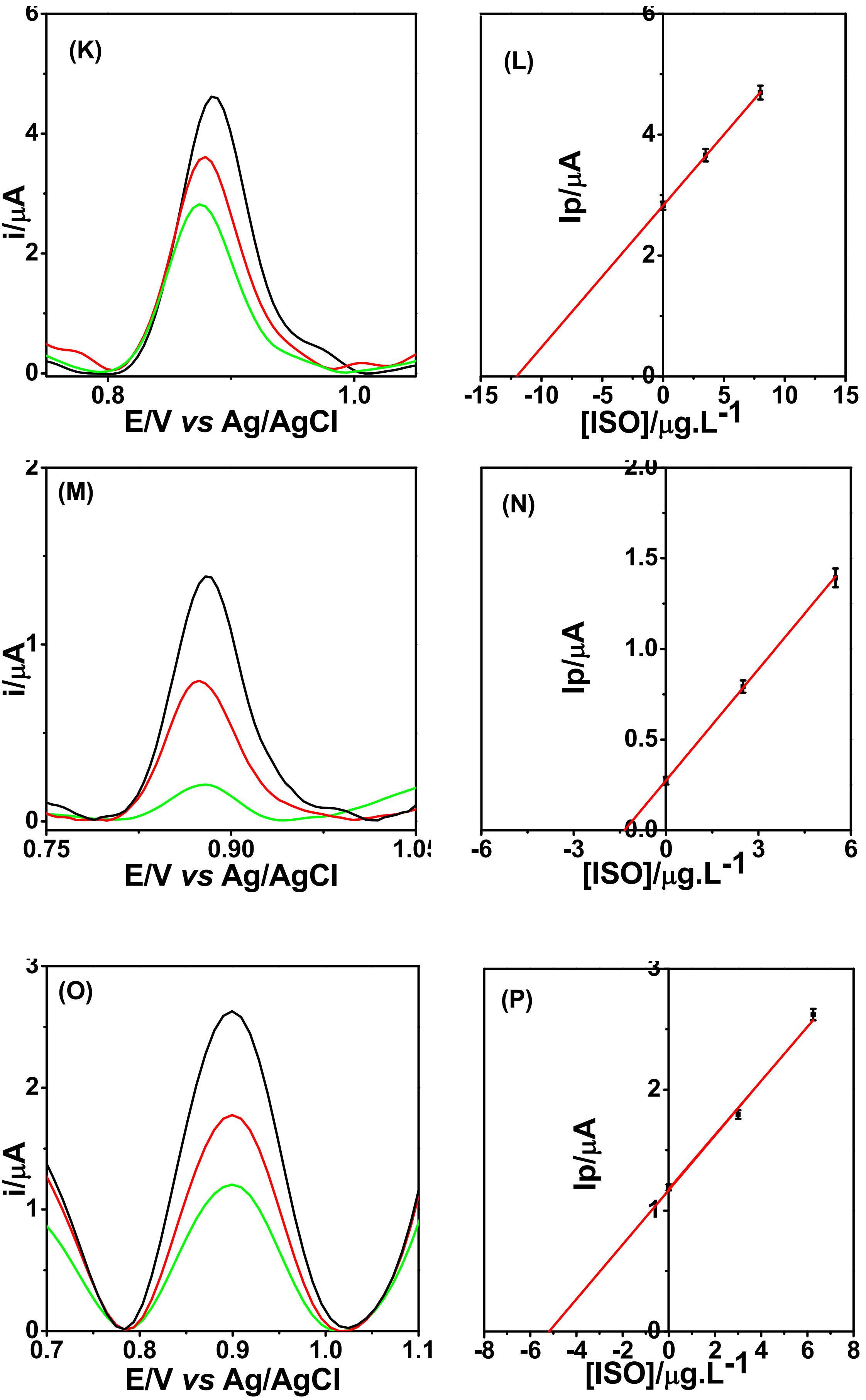
(K)
i/µA
E/V vs Ag/AgCl
(L)
Ip/µA
[ISO]/µg.L-1
(M)
i/µA
E/V vs Ag/AgCl
(N)
Ip/µA
[ISO]/µg.L-1
(O)
i/µA
E/V vs Ag/AgCl
(P)
Ip/µA
[ISO]/µg.L-1

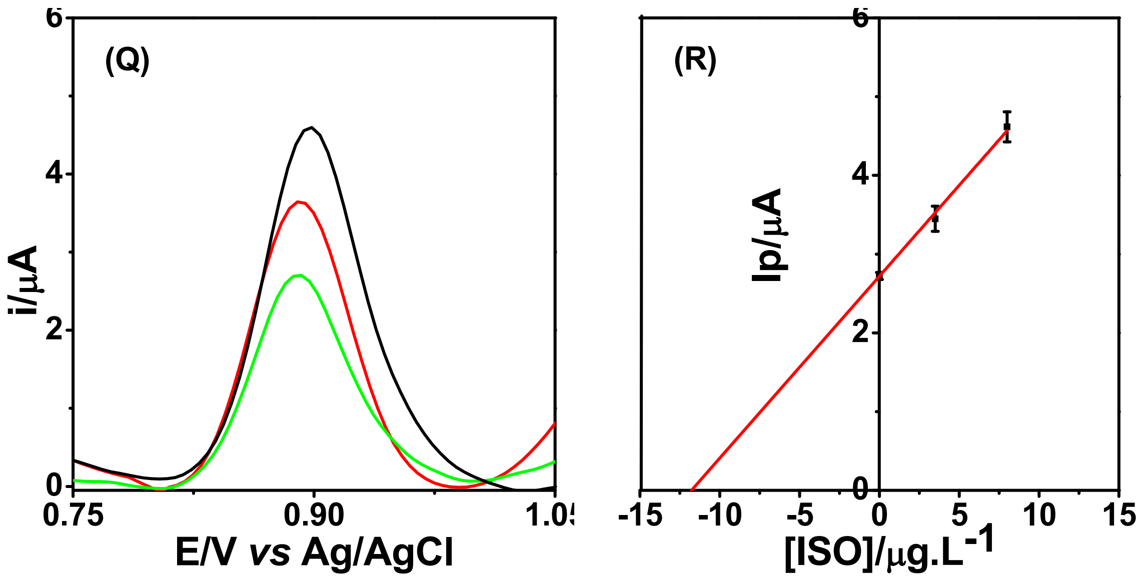

Figure S7 - Recovery of spiked ISO by VPD in the presence of A) 1.50 µg L^{-1} in surface water; B) Ip vs. ISO concentration in surface water; C) 5.75 µg L^{-1} in surface water; D) Ip vs. ISO concentration in surface water; E) 12.00 µg L^{-1} in surface water; F) Ip vs. ISO concentration in surface water; G) 1.50 µg L^{-1} in tomato extract; H) Ip vs. ISO concentration in tomato extract; I) 5.75 µg L^{-1} in tomato extract; J) Ip vs. ISO concentration in tomato extract; K) 12.00 µg L^{-1} in tomato extract; L) Ip vs ISO concentration in tomato extract; M) 1.50 µg L^{-1} in human plasma; N) Ip vs ISO concentration in human plasma; O) 5.75 µg L^{-1} in human plasma; P) Ip vs ISO concentration in human plasma. Q) 12.00 µg L^{-1} in human plasma; R) Ip vs ISO concentration in human plasma (*n=3* for all measurements).

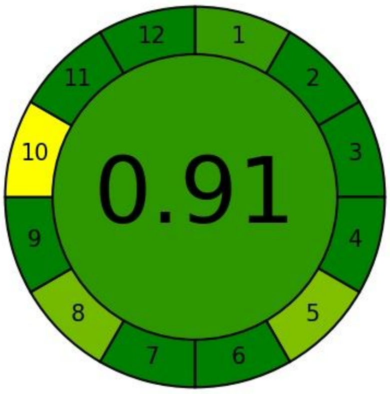

Figure S8 – AGREE method of greenness evaluation.

Table S1 - DPV optimization study for ISO at the AgAu NSs/SiO_2/NF/GCE platform.

Parameter	Studied range	Fixed condition
Scan rate / mV s^{-1}	10 - 50	30
t_{pulse} / s	0.005 -0.02	0.008
$E_{amplitude}$ / mV	10 - 100	70

Table S2. Comparative interference response (*n* = 3) tested in the presence of a 5-fold concentration of different potentially interfering species.

Potential interferents	Relative Error (%)
**Cd^{2+}**	-3.39
**Pb^{2+}**	+1.75
**Zn^{2+}**	-1.91
**Cu^{2+}**	+0.38
**Ascorbic Acid**	+1.36
**Uric Acid**	+14.02
**Cafeic Acid**	+1.12
**Citric Acid**	-0.56
**Diuron**	-3.82
**Pirimicarb**	-11.21
**Bisphenol**	-3.00
**Roxarsone**	-4.40
**Gliphosate**	+4.94
**Carbofuran**	-3.25

Table S3 - Recovery values relative to the standard addition method for ISO determination.

Sample	Parameters			
Water	Added (μg L^{-1})	**1.50**	**5.75**	**12.00**
	Found (μg L^{-1})	1.24	5.33	11.44
	RSD (%)	1.63	3.80	4.66
	Recovery (%)	83.0	92.7	95.3
Tomato Extract	Added (μg L^{-1})	**1.50**	**5.75**	**12.00**
	Found (μg L^{-1})	1.30	5.42	12.05
	RSD (%)	1.56	3.73	4.76
	Recovery (%)	86.5	94.3	100.4
Simulated plasma	Added (μg L^{-1})	**1.50**	**5.75**	**12.00**
	Found (μg L^{-1})	1.35	5.28	11.77
	RSD (%)	4.83	3.83	12.69
	Recovery (%)	89.8	91.8	98.1
